# Replacement of Missing Anterior Teeth in a Patient with Chronic Mouth Breathing and Tongue Thrusting

**DOI:** 10.1155/2013/759162

**Published:** 2013-12-29

**Authors:** Satheesh B. Haralur, Ali Saad Al-Qahtani

**Affiliations:** Department of Prosthodontics, College of Dentistry, King Khalid University, Abha 61417, Saudi Arabia

## Abstract

The loss of anterior teeth has serious functional, esthetic disabilities, in addition to compromising the patients' quality of life. Various etiologies can be attributed to the anterior tooth loss, including trauma, caries, and periodontal diseases. The chronic mouth breathing due to nasal adenoids is known to enhance the gingival and periodontal diseases. The dental literature proves the association of nasal breathing, tongue thrusting, and anterior open bite. Arch shape and tooth position are primarily determined by the equilibrium of the forces from tongue and perioral musculature. Increased force from tongue musculature in the tongue thrusting patient leads to flaring of anterior teeth, making them susceptible for periodontal and traumatic tooth loss. Replacement of the anterior teeth in this patient will also help in restoration of anterior guidance, which is critical for the health of temporomandibular joint, posterior teeth, and musculature.

## 1. Introduction

Loss of anterior teeth has major detrimental social implications for the sufferer and significantly affects the normal social integration. Loss of anterior teeth is from multifactorial origin ranging from trauma, periodontal disease, dental caries, and persisting oral habits. If preventive treatment is not provided at an early age, they continue to persist up to adult age. Potential detrimental effects and management of these persisting oral habits are often overlooked by the oral health professionals. The excessive lymphoid tissue proliferation with chronic allergy and infection lead to obstruction of the nasal airway, resulting in chronic mouth breathing [1]. Studies have shown the strong correlation between mouth breathing as an etiological factor for tongue thrusting and anterior dental open bite [2]. The long existing malocclusion leads to having the tongue filling the space and results in loss of lip and muscle tonus. The disturbed balance between the force of the tongue and oral musculature leads to anterior migration of teeth. The exposed, flared teeth are highly susceptible for traumatic damage. The mouth breathing is also considered as one of the predisposing factors for initiation of periodontal disease [3, 4]. The chronic mouth breathing patient with high incidence of periodontal disease tend to lose the anterior teeth in their early age. Anterior dental open bite and loss of anterior teeth impede the emission of dental-alveolar sounds [5, 6]. The absence of anterior guidance prolongs the disocclusion time and predisposes the patients for temporomandibular disorders [7]. There is a paucity of dental literature regarding the effect of chronic mouth breathing and tongue thrusting and its prosthetic management. The well designed prosthesis can completely rehabilitate the aesthetic, functional, and phonetic debilitation. The objective of this case report is to explain the clinical prosthetic rehabilitation of the chronic mouth breathing and tongue thrusting patients with loss of maxillary and mandibular anterior teeth.

## 2. Case Presentation

**Figure 1:**
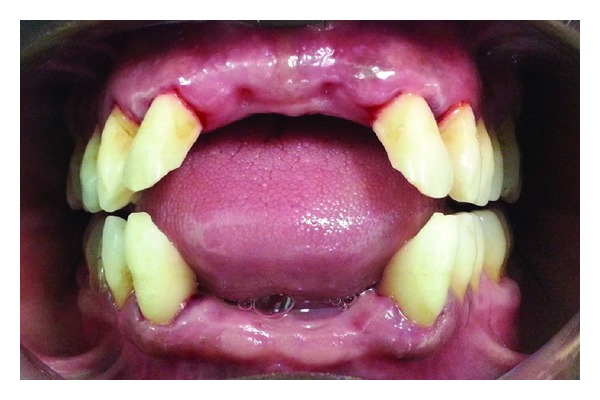
Patient presenting with loss and flaring of teeth and tongue occupying the space.

A 35-year-old male patient visited the King Khalid University dental clinic for the replacement of both maxillary and mandibular anterior missing teeth. The patient was extremely unhappy with existing transitional, removable partial denture due to its poor stability, especially during speech and mastication. He gave a history of teeth extraction six months before due to mobility. He also reported on chronic mouth breathing due to enlarged adenoids and blocked nasal breathing, which was surgically treated a year back. The patient gave a graphic history of continued, progressive labial flaring of anterior teeth that subsequently became mobile to be extracted. On examination, it was observed that the patient had lost both maxillary central incisors and all four mandibular incisors (Figure 1). The adjacent maxillary lateral incisors showed slight labial flaring and grade 1 mobility; intraoral periapical X-ray confirmed approximately 20–25% alveolar bone loss. Routine TMJ examination showed no signs of pathology. The patency of nasal airways were checked by requesting to close the lip together and occluding one naris with the index finger alternatively. Ask the patient to breathe normally through open naris. The tongue movements were within normal limits. The evaluation of swallowing process without the removable prosthesis showed slight space between posterior teeth and minor hyperactivity of orbicularis oris and mentalis muscles. On complete evaluation of clinical signs symptoms along with clinical examination, it was diagnosed as loss of maxillary and mandibular anterior teeth due to combined effect of periodontal diseases, chronic mouth breathing, and tongue thrusting.

The treatment objectives were to replace the missing maxillary and mandibular central incisors along with rehabilitation of tongue thrusting habit. Treatment options to replace the missing anterior teeth was discussed with the patient, including the implant supported fixed prosthesis. The tooth that supported conventional fixed partial denture was selected according to the patient desire. The potential abutments were evaluated by the clinical and radiological examination to ascertain periodontal and pulpal health. The intentional root canal treatment was performed on maxillary right lateral incisors to gain common path of insertion. The bilateral canines and lateral incisors were the abutment for maxillary central incisors, while bilateral canines were selected as abutments for mandibular incisors [8]. The absence of gross soft or bony defect in the corresponding residual ridge of missing teeth was also favorable for tooth that supported fixed partial denture.

**Figure 2:**
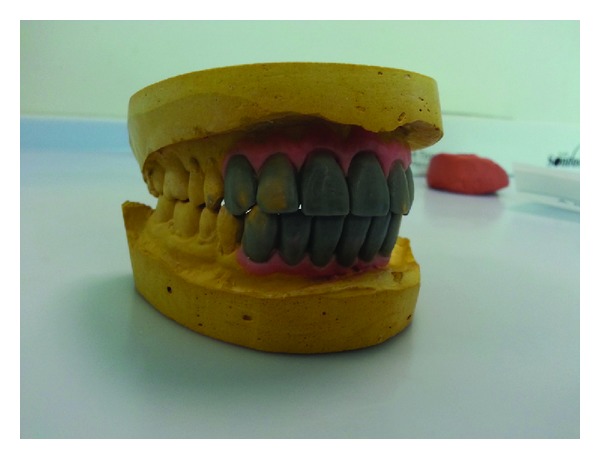
Diagnostic wax up of the diagnostic cast.

The diagnostic casts were made from irreversible hydrocolloid impression materials, with the help of face bow records mounted on a semiadjustable articulator. The existing removable denture had more teeth to compensate the slightly larger residual ridge space. To ascertain the esthetic outcome and patient response, the diagnostic wax up for the missing teeth structure was done (Figure 2). To obtain the acceptable esthetic proportion between the teeth, it was decided to distribute the excess edentulous area between abutment and pontics.

The autopolymerized acrylic denture base for the edentulous area was fabricated on dental cast; the modelling plastic compound was used to make the occlusal rim. The vertical height of the occlusal rims was adjusted according to the anatomic landmarks, visibility, and phonetics. The modelling plastic compound was softened and the patient was asked to perform all physiological muscle functions by sucking, swallowing, and phonetics. The recontoured modelling compound gave the exact tooth position and inclination according to the neutral zone [9].

**Figure 3:**
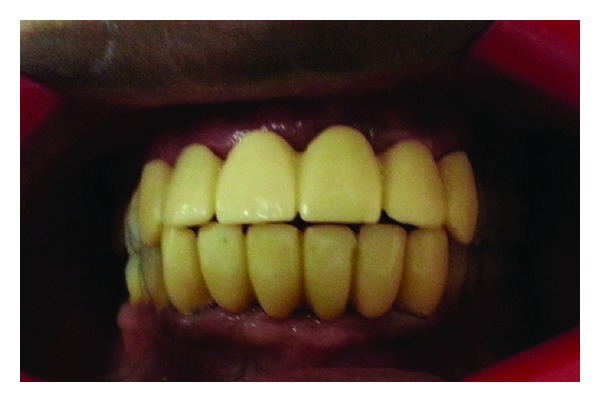
Refined and finalized provisional restoration.

Split putty indexing was made to guide the dental technician on the exact position and inclinations of pontics during fabrication of provisional restoration. The abutments were prepared for metal ceramic retainers; the definitive impression was made with heavy body-light body silicone impression materials. The poly ethyl methacrylate provisional fixed partial dentures were fabricated with indirect method. The provisional bridges were cemented with noneugenol temporary luting cement after minor correction for aesthetics, phonetics, and occlusion (Figure 3). The Patient was given an instruction to follow the tongue neuromuscular sensory stimuli by brushing and resistance to regain the desirable motor response [10]. The patient was recalled after 24 hours to evaluate the patient opinion, gingival health, and comfort. The patient was given a scheduled appointment for six weeks to evaluate the overall satisfaction of esthetics, phonetics, and function. The continuous required minor adjustments were made to the provisional bridge during evaluation period. The provisional restorations with acceptable anterior guidance, esthetics, phonetics, and comfort were replicated to minute detail in permanent restoration.

**Figure 4:**
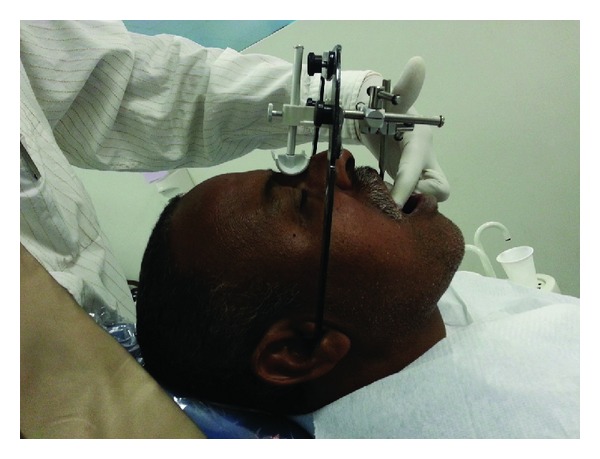
Face bow transfer for mounting working cast.

**Figure 5:**
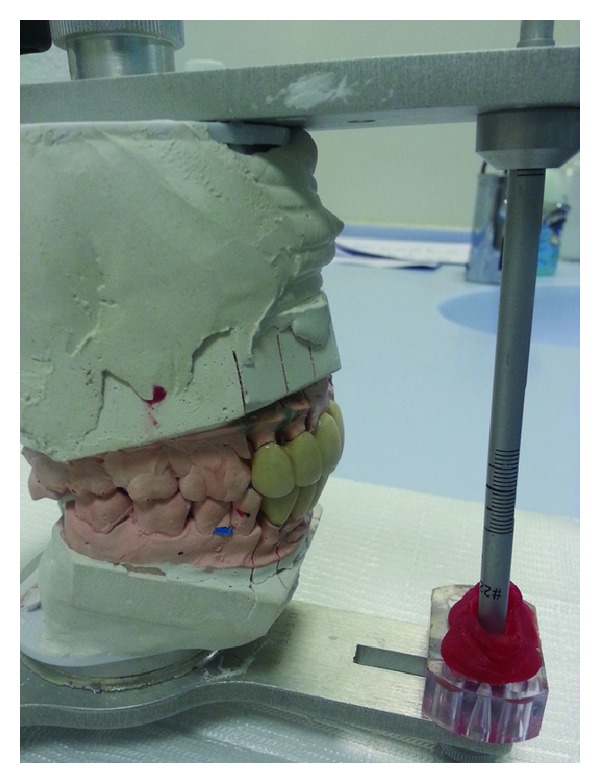
Customised incisal table.

**Figure 6:**
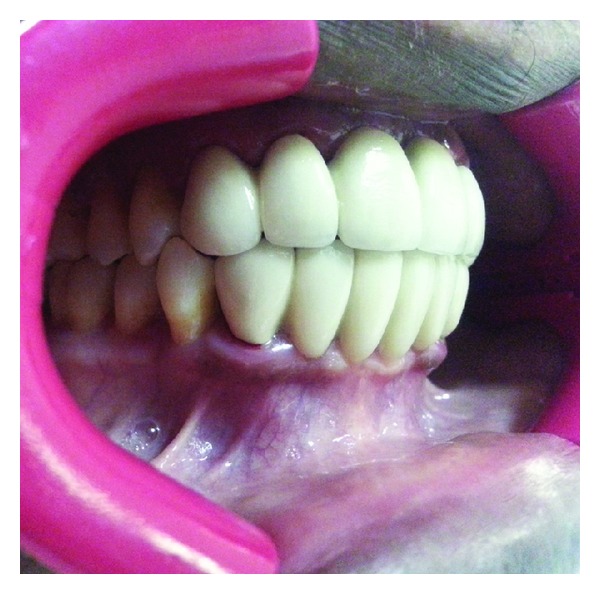
Final prosthesis.

**Figure 7:**
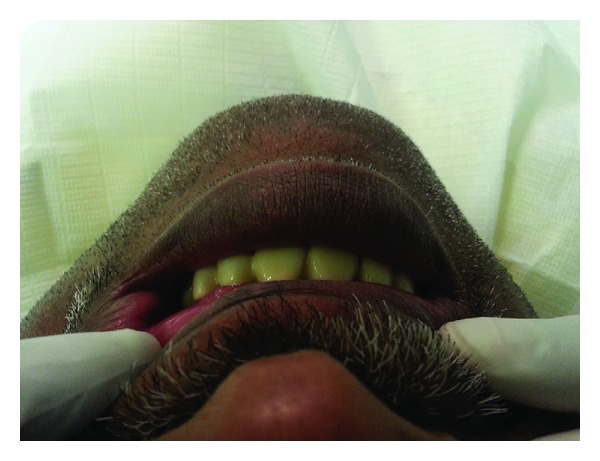
Establishment of proper lip position over the maxillary incisors.

**Figure 8:**
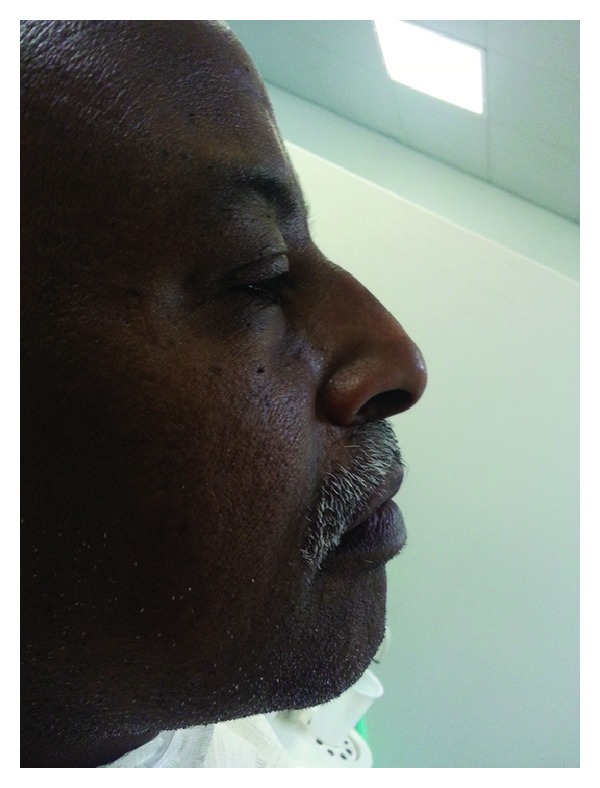
Reestablished lip support and lip competency.

The alginate impression with the cemented provisional restoration was made, and dental cast was mounted on the semiadjustable articulator with face bow transfer (Figure 4). The autopolymerized acrylic customized incisal guidance table was prepared on the semiadjustable articulator from the mounted casts (Figure 5). The customized incisal table enabled the dental technician to replicate the exact anterior guidance in the final restorations. The putty indexing of the provisional restorations was used to remake the labial contour, shape, and inclination in the final metal ceramic fixed partial denture. The final prosthesis was cemented with the glass-ionomer type-I luting cement after required occlusion refinements (Figures 6, 7, and 8).

The patient was recalled after one week to evaluate the residual cement, gingival health, and occlusal integrity. The patient was monitored during the first three months with a two-week-interval, followed by a month intervening three months. The patient showed a remarkable improvement in speech and controlling the tongue thrusting. He was highly satisfied with the aesthetic outcome of the prosthesis.

## 3. Discussion

The loss of anterior teeth compromises the patient's quality of life, its replacement will help to restore the functions of mastication, speech, and aesthetics. Evaluation of the history and disease course to understand the reason for tooth loss is vital to treatment outcome. Upper airway obstruction that lead chronic mouth-breathing may cause anterior open bite. The studies reported the existence of the strong association between chronic mouth breathing, dental anterior open bite, and tongue thrusting [10, 11]. Though some researchers believe the tongue merely occupies the space created by the malocclusion [12]. The studies have also reported that the tongue thrust leads to significant increase in maxillary anterior teeth proclination [13]. The increased incidence of gingival inflammation and periodontitis is observed in the chronic mouth breather, hastening tooth loss [3, 4, 14, 15]. The associated lip incompetency creates significant imbalance in force between the tongue and perioral musculature, resulting in progressive flaring of incisors [16]. The prosthetic rehabilitation should carefully consider all the involved factors to address them individually.

Persistent tongue thrusting lead to failure of orthodontics, malocclusion, and instable removable prosthesis. The replacement of the teeth in the neutral zone will help in stabilizing the tooth position and restore proper lip support. It will also help in eliminating the detrimental lateral force from tongue musculature on the supporting abutment [17, 18]. The neutral zone determination is followed mainly in poorly supported complete denture fabrication. It is important to reestablish the tooth position in neutral zone during simultaneous replacement of maxillary and mandibular incisors in a patient with tongue thrusting. The reestablishment of incisor vertical overlap help to restore the missing anterior guidance. The research has shown that the optimum anterior guidance is vital for the health of both posterior teeth and temporomandibular joint [7, 19]. The replacement of missing anterior teeth morphological characteristics and their relation to oral structure are also important for rehabilitation of phonetics. The quality of the prosthesis, especially the reduced stability, can compromise the speech production; prolonged use of improper prosthesis harm the adjustment to the new prosthesis [20]. The tongue dysfunction habits are required to be treated for the long term positive prognosis of the prosthesis [21]. The tongue is remarkable in its ability to adapt to changes in the surroundings; the neuromuscular facilitation stimulation enable the faster tongue adaptation. Brushing and pressure application act as sensory stimuli to reduce the flaccidity of the tongue. The well designed prosthesis and meticulous followup care will completely rehabilitate patients with tongue thrusting with anterior tooth loss.
